# Editorial Note

**Published:** 1926

**Authors:** 


### Editorial Note.

**A Country Hospital for Bristol Cripples.**

FOR the past five or six years the inadequacy of Bristol's provision for the treatment of crippling disease, especially in children, has been troubling the minds of many people. Good work has been and is still done by the Orthopædic Hospital, the Children's Hospital, and by the two big general hospitals ; and to this must be added the care for patients with surgical tuberculosis provided by the Municipal Council, who have had the great disadvantage of possessing no premises of their own for the purpose. It is, in fact, this lack of means for treating the patients for whose welfare they are by law responsible that has brought the City Health Committee to formulate proposals for the development of an orthopædic hospital at Frenchay Park, an estate that was bought by the city on favourable terms when it came into the market.

Now, side by side and always in touch with this municipal movement, there has developed a voluntary movement, embodied in the Crippled Children's Society, of which Miss F. M. Townsend is President. This Society has for some years done excellent work on behalf of the Crippled Children of Bristol, including among these children with heart disease and other affections that are indeed "crippling," though not always so described. It has recently become united with the Orthopædic Hospital in Redland, and the two bodies have decided to build a country school-hospital as soon as they have funds enough. They made proposals for union with the municipal hospital, which were examined by the Ministry of Health, but as the Ministry reported in favour of a plan by which the voluntary body would provide a kind of convalescent home for the patients from the city's Orthopædic Hospital and for other patients, the hopes of union have been somewhat dashed. The Voluntary Committee, also, thinks that some site better than the Frenchay one might be found, and indeed has been looking carefully into the possibilities of a country site with a protected southerly aspect.

There can be no doubt that the medical profession will support a policy of union. Their wish for united effort in the treatment of crippling disease in childhood has found expression again and again, and Professor Hey Groves has exerted himself greatly to bring it about. Neither can there be any two opinions about the question of a site. It ought to be the best that the neighbourhood offers, and it must be large enough to allow of the addition of new blocks. The big voluntary hospitals of the city can hardly hope to extend their in-patient provision on their present sites, and much has already been said of the possibility of a gradual migration to the outskirts of the city. It would be a thousand pities if anything were to ruin the city's chance of uniting forces in the near future, and we earnestly hope that the long view will prevail.

